# Smart and Active Food Packaging: Insights in Novel Food Packaging

**DOI:** 10.3389/fmicb.2021.657233

**Published:** 2021-07-09

**Authors:** Hamed Ahari, Solmaz P. Soufiani

**Affiliations:** Department of Food Science and Technology, Science and Research Branch, Islamic Azad University, Tehran, Iran

**Keywords:** active food packaging, smart food packaging, biopolymers, shelf life, nanomaterial

## Abstract

The demand for more healthy foods with longer shelf life has been growing. Food packaging as one of the main aspects of food industries plays a vital role in meeting this demand. Integration of nanotechnology with food packaging systems (FPSs) revealed promising promotion in foods’ shelf life by introducing novel FPSs. In this paper, common classification, functionalities, employed nanotechnologies, and the used biomaterials are discussed. According to our survey, FPSs are classified as active food packaging (AFP) and smart food packaging (SFP) systems. The functionality of both systems was manipulated by employing nanotechnologies, such as metal nanoparticles and nanoemulsions, and appropriate biomaterials like synthetic polymers and biomass-derived biomaterials. “Degradability and antibacterial” and “Indicating and scavenging” are the well-known functions for AFP and SFP, respectively. The main purpose is to make a multifunctional FPS to increase foods’ shelf life and produce environmentally friendly and smart packaging without any hazard to human life.

## Graphical Abstract

Novel food packaging comprises several technologies.

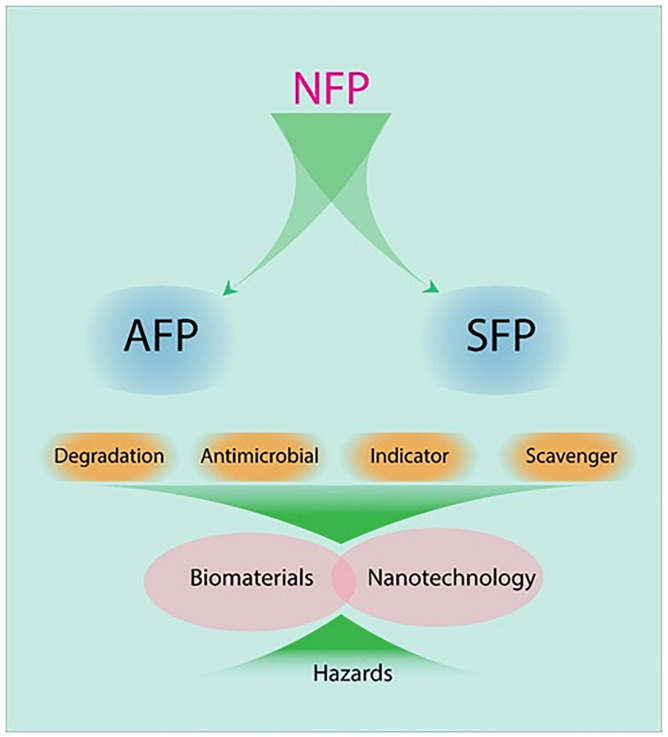

## Highlights

- Novel food Packaging comprises Active and Smart food packaging.
- Nanotechnology plays a vital role in novel packaging systems.
- Food’s shelf life is prolonged under Active packaging systems.
- Consumers can be informed about the quality of foods by smart packaging systems.
- Novel packaging systems may hazards human health.

## Introduction

Basically, food packaging is one of the vital steps in the food industry, so that a suitable packaging not only attracts the customer’s attention but also keeps the products at the highest possible level of nutrition and quality (Gupta and Dudeja, 2017). There are three levels of packaging, namely, primary packaging, secondary packaging, and tertiary packaging (Grönman et al., 2013). Primary packaging is the coating/film that directly encloses the food and communicates with the food. Primary packaging is the main layer affecting the quality of foods due to its direct contact with the materials. Secondary packaging covers the products packaged by the primary packaging. Tertiary packaging is the outer packaging employed for bulk handling, distribution, and storage. Despite these three layers of packaging, it is possible that food safety is not guaranteed (Muncke, 2014).

Transporting easily, protecting food quality, maintaining food integrity, keeping away from harmful particles and chemicals, and preventing bacterial growth and pests are the major benefits of food packaging including primary, secondary, and tertiary packaging (Robertson, 2014). Packaging also gives consumers necessary information about the products via labeling. The name of products, the list of ingredients, the way of consumption, the price, and the expiry date are the main information reported by labeling (Roche, 2016).

Microbial spoilage and its metabolism and oxidation are the principal reasons for many food deterioration, such as bananas, tomatoes, pears, apples, mangoes, and kiwifruit, from production and transportation till storage and marketing (Petruzzi et al., 2017). Communication, protection, containment, and convenience are the most common features of traditional food packaging (TFP). TFP is a typical system providing just physical support and food protection against stimuli and environments in the packaging process, distribution, transportation, and storage (Lloyd et al., 2019). In general, an effective TFP often helps preserve foods and is just a nonfunctional physical barrier against chemical, physical, and microbial damage (Robertson, 2019). It is also estimated that TFP generates tons of waste annually. However, due to the progress of technology and modern life, demands for a healthy and high-quality food product, easy transportation, and especially long shelf life are increasing; therefore, TFP systems are not able to meet the needs of the consumer, and hence, an appropriate alternative is necessary.

**FIGURE 1:**
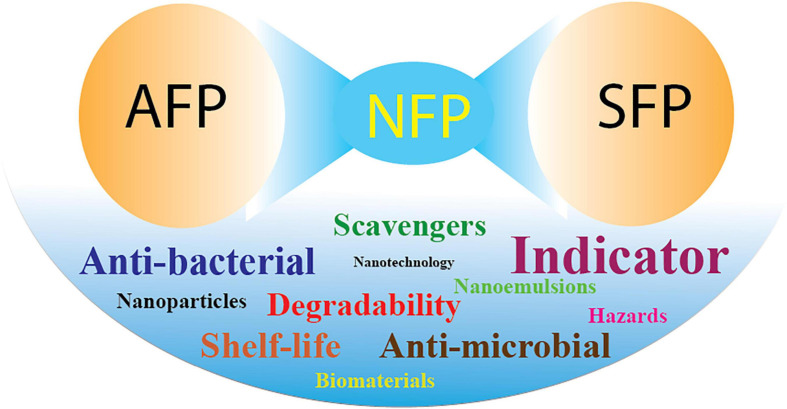
New types of food packaging systems and their performances.

Considering primary packaging, it is required to develop novel food packaging (NFP) systems by employing different biomaterials and techniques, embedding sensors and indicators, different functions, biodegradable materials, nanotechnology, essential oils, and plant extracts, while maintaining the quality and nutrition and improving the shelf life of food; the environmental effects of the package reduced food rationing (Figure 1; Zhang and Zhao, 2012; Liu et al., 2020; Zhang et al., 2021). The strengths of NFP, also known as active, smart, and green technologies, are the lack of inactivity and negative inactivity between packaging and food components, long-term performance, prevention of food spoilage, and enhancement of consumer health, and these can be considered as the ultimate goal in the future for food packaging technology (Lone et al., 2016).

As can be seen from Figure 1, there is a difference between smart food packaging (SFP) and active food packaging (AFP). SFP monitors the condition of packaged foods to provide information about their quality and nutrients, before consumption, while in AFP, there are some mechanisms to control microbial growth, moisture, and oxidation.

In general, SFP and AFP are two promising packaging systems in the food industry (Table 1). SFP allows the consumer to detect changes in food quality parameters over time due to the presence of certain substances in its matrix, which act as indicators. As can be seen in Table 1, packaging systems, depending on foods, can be designed as coating or films (thick or thin). Both of them can be engineered as AFP or SFP. Employing this system in FPS will increase food shelf-life by controlling microorganisms’ growth, food freshness, appropriate color, desired smell, customer satisfaction, and so on.

**TABLE 1:** a short description of some NFP systems.

Food packaging system	Type of FPS AFP - SPF	Employed materials	Type of nanomaterial	Purpose	Results	References
Film	AFP	Polyvinyl Chloride	–	Degradation in soil in present of Tenebrio molitor larvae	• T. molitor larvae are capable of performing • broad depolymerization/biodegradation.	Peng et al., 2020
Film	AFP	Chitosan, gelatin, polyethylene glycol	Ag NP	Biodegradation	• The shelf life of the fruit extended for additional two weeks. • Nano-Ag enhanced the mechanical properties and reduced transparency. • The film is appropriate for FPS.	Kumar et al., 2018
Film	SFP	Polylactide and polyhydroxybutyrate	Colorants	Color-based indicator	• Color changes depicted the life time of the materials. • changes in the samples color under due to UV, thermooxidation, and weathering • The system is biodegradable.	Latos-Brozio and Masek, 2020
Film	SFP	Polyolefin elastomer (POE)	Potassium permanganate, nanoclay and nanosilica	Ethylene scavenger	• The film showed improvement in mechanical properties, larger Ethylene scavenging, and lower Water Vapor Permeability compared to the neat POE film. • Ethylene absorption efficacy enhanced at higher level of impregnated NP because of their higher potassium permanganate concentration. • The optimized film could extend the shelf life of bananas up to 15 days.	Ebrahimi et al., 2021
Film	AFP	Polyhydroxybutyrate	Eugenol	Degradation in various soil type (agricultural, landfill and sandy)	• Films in agricultural soil showed a higher biodegradation due to high fungi load. • The phosphorus availability, soil acidity, moisture and crystallinity of polymer were critical factors in evaluation of the differences in biodegradation rates microbial growth. • Eugenol enhanced the polymer crystallinity and decreased the mechanical properties.	Rech et al., 2020
Film	AFP	gelatin-chitosan	TiO_2_-Ag NP	antimicrobial	• The addition TiO_2_-Ag in the film enhanced the interaction between components and notably enhanced the water solubility. • When the concentration of TiO_2_-Ag increased to 0.5%, the best antibacterial ability was observed. • The addition TiO_2_-Ag to the film, decreased the tensile strength of the film.	Lin et al., 2020
Film	AFP	Cellulose nanofiber	Carbon and Ag NP	Antimicrobial	• The film were evaluated against food pathogens, *S. aureus and E. coli*. • 140–450 ppm was the optimum concentration of Ag NP to inhibit the growth of *S. aureus and E. coli.*	Sobhan et al., 2020
Film	SFP	Cellulose	TiO_2_ NP	Oxygen-scavenger	• The film could scavenge oxygen at the rate of 0.017 cm^3^ O^2^ h^–1^ cm^–2^ during 24 h.	Mills et al., 2006
Film	SFP	Gluten	chlorophyll/polypyrrole	Conductivity and color indicator	• Adding chlorophyll and polypyrrole increased the opacity and tensile strength of films. • Chlorophyll did not affect the antibacterial property against E.coli while polypyrrole did vice versa. • The film can be employed as SFP because of changes in its conductivity and color during storage.	Chavoshizadeh et al., 2020
Film	SFP	Cellulose/chitosan	Carrot anthocyanin	pH-responsive indicator	• The water solubility and swelling enhanced by addition of carrot anthocyanin into the film. • The pH indicator depicted a clear color changes from pink to khaki at various pH values between 2–11. • Based on the stability tests, the pH indicator showed an acceptable color stability within storage for one month at 20•°C. • The addition of carrot anthocyanin showed no effect on the super-molecular and chemical structure of the films.	Ebrahimi Tirtashi et al., 2019
Film	AFP	Hydroxyethyl cellulose	ZnO NP	Antimicrobial	• The film stopped *S. aureus* and *E.coli* bacteria growth. • ZnO addition improved the mechanical properties of the films.	El Fawal et al., 2020
Coating	SFP	Chitosan	ZnO NP	Antimicrobial	• ZnO NP at ≥0.0125% stopped *E. coli* growth at 37°C or 10. • The coating notably decreased *E. coli* population on white cheese at 4°C or 10. • The coating increased white cheese color but didn’t affect a_*w*_.	Al-Nabulsi et al., 2020
Film	SFP	low density polyethylene (LDPE)	curcumin	A hydrophobic ammonia sensor	• The LDPE-curcumin composite film was sensitive to ammonia • The film depicted light yellow to light brown color changes during the storage time in case of beef storage at 4 •C. • Color changes means enhancement in TVB-N contents of the meat samples.	Zhai et al., 2020
Coating	AFP	Polyethylene	Carvacrol	Antimicrobial	• The coating decreased the bacterial growth on packaged chicken surfaces. • coated surfaces showed low bacterial attachment.	Alkan Tas et al., 2019
Coating	SFP	–	PdCl_2_–CuSO_4_, carbon powder	Ethylene scavenger	• CuSO_4_ and PdCl_2_ addition enhanced ethylene removal efficacy. • Ethylene scavenging capacity of the film can be regenerated. • The film could inhibit ethylene production and increased the shelf life of broccoli.	Cao et al., 2015
Film	SFP	LDPE, HDPE and PP	Carvacrol	Microbial activity-based indicator	• Employing several film layers • Prediction of the expected shelf life during food storage.	Vilas et al., 2020
Film	SFP	EMCO and ATCO	sodium carbonate,	Oxygen and carbon dioxide	• Color seemed better in all treated strawberries. • Scavenging oxygen and carbon dioxide increased the shelf life of strawberries. • The use of these scavengers depicted slowed consumption of oxygen and carbon dioxide accumulation.	Aday et al., 2011
Film	SFP	cellulose	Iridescence (as a color)	color humidity indicator	• When the film was exposed to the water or high relative humidity, a shift in the film’s color observed from blue-green (dry state) to red-orange (wet state). • The art of color change depends on the film thickness (40 m: 1–3 min).	Zhang et al., 2013
						

As Table 1 shows, several technologies including nanomaterials (Kaur et al., 2020), biomaterials (Asensio et al., 2020), nanoemulsion (Moghimi et al., 2017), microbiology (Jaworska et al., 2020), and food science can play a vital role in NFP. It is hypothesized that the role of these technologies and the way they perform in NFP are still of interest to scientists. Based on our survey, numerous studies considering new food packaging systems have been published. Giving scientists a comprehensive review of NFP would be beneficial. The current study was carried out to provide a comprehensive review about the NFP systems including AFP and SFP by covering the main functions, employed biomaterial, and the role of nanotechnology and finally discussing the related hazards.

## NFP Systems

### Degradability of Food Packaging

Considering AFP/SFP, various functions and features are designed by scientists for new packaging (Table 1). For example, regarding environmental issues, leaving the residue of packaging in the environment causes pollution and hazards to human and animal life (Marsh and Bugusu, 2007). Food packaging utilizes various materials to protect foods, of which a high percentage are nondegradable and remain in the environment for years. Metal and glass as food containers are the most well-known and traditional materials in FPS (Marsh and Bugusu, 2007).

As can be inferred from Table 1, the presence of polymers, such as polystyrene, polyvinylchloride, polyethylene, polyethylene terephthalate, polypropylene, and polyamide, in food packaging systems provided more satisfaction to customers in the viewpoints of ease of use, appearance, and transparency (Stoica et al., 2018). Furthermore, these polymers showed good availability, lower price, good water vapor permeability, good mechanical properties (tensile strength and shear strength), and good gas barrier properties (oxygen and carbon dioxide) (Pawar and Purwar, 2013; Stoica et al., 2018). They can also be natural (e.g., gelatin-chitosan) or synthetic (e.g., polycaprolactone). In spite of this, contamination and environmental pollution issues still remain unsolved (Moore, 2008; Williams and Wikström, 2011).

“Environmentally friendly” is a new term defined in NPS. According to European law, biopolymers and bioplastics must be biodegradable, especially in terms of composting, so they can act as soil softeners and fertilizers. This issue has been indicated in Table 1. Many researchers have evaluated the biodegradability of the prepared packaging by burying it in soil (Table 1). However, some natural plastics based on natural monomers may lose their biodegradability through chemical modification of the polymerization process. Materials that ensure not only the nutrition and maintenance of the product (from production to consumption) but also their release into the environment do not pose a risk to the environment and decompose over time and, as one of the primary goals in the SFP and AFP, were taken into consideration (Siracusa et al., 2008).

There are a variety of biomaterials which are classified according to their source. (i) Natural polymers have attracted the attention of scientists due to their natural source and higher-degradability features. Chitosan, polysaccharide, starch, alginate, and gelatins are examples of these biomaterials which are nontoxic and environmentally friendly (Malhotra et al., 2015). (ii) Biomaterials generated by the activity of microorganisms such as bacterial cellulose, polyhydroxybutyrate (PHB), and xanthan are suitable for various medical and industrial applications (Rehm, 2010). (iii) Synthetic products are derived from natural sources of biomass and oil (biopolyester or lactic acid) or from a polymerization process and renewable monomers such as polyethylene terephthalate (PET) (Ali et al., 2006; Hacker et al., 2019). Basically, using the current petroleum-based polymers such as PET and polyamide in FPS is still the only way of packaging production (Ferreira et al., 2016; Mishra, 2018; Bumbudsanpharoke and Ko, 2019). Plastic waste is a global problem, and the doubling of global plastic production over the next decade is expected to have a major adverse effect on the environment due to its lack of environmental degradability (GreenFacts, 2020).

Many plastics are mixtures of synthetic components such as polymers and additives to improve the functional properties of the final product and expand the scope of application. In this regard, numerous studies have been conducted to produce new packaging with the aim of shortening the residence time in the environment using renewable resources and biodegradable materials. Along with biodegradability, there are other properties which must be considered and fulfilled. Biodegradable packaging from biopolymers requires some water solubility to promote degradability. However, at the same time, hydrophilic property decreases mechanical and barrier properties. This contradiction has also encouraged the development of some NFP systems. These features are important to modify and control the barrier and mechanical properties which are related to the polymeric packaging material structure (Boyle et al., 2019).

**FIGURE 2:**
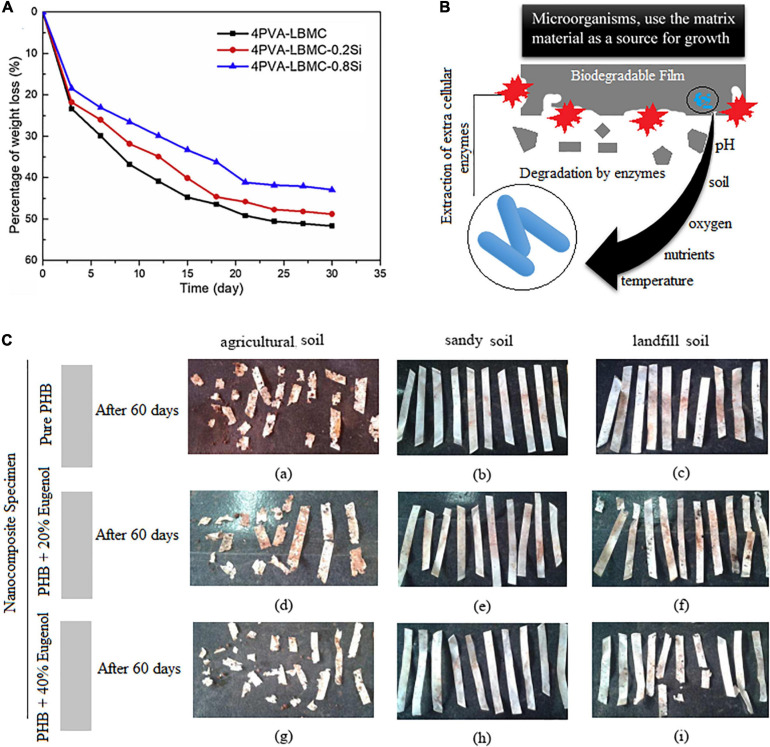
**(A)** Biodegradation of the blend films in the soil [reproduced with permission from Zhang J. et al. (2020)]. **(B)** The mechanism of biodegradation of the film matrix. **(C)** Pure polyhydroxybutyrate (PHB) films containing eugenol after 60 days of biodegradation. (a–c) Pure PHB. (d–f) PHB films containing 20% of eugenol. (g–i) PHB films containing 40% of eugenol [reproduced with permission from Rech et al. (2020)].

In a research done by Goudarzi and Shahabi-Ghahfarrokhi (2018), a photodegradable bio-nanocomposite starch/TiO_2_ was produced as a food packaging material using photochemical-based reactions. The prepared film showed good degradability under UV exposure and reduction in the mechanical and barrier properties. It was reported that UV rays lowered the hydrophobicity of the films and that enhanced duration of exposure negatively reduced Young’s modulus and tensile strength. Degradation behavior in water/in soil/on the earth is one of the main characteristics of food packaging materials. Addition of nano-silica to biopolymeric films such as polyvinyl alcohol/liquefied ball-milled chitin showed good degradation in soil (Figure 2A; Zhang J. et al., 2020). Employing alkali hydrolysis and microbial/bacterial attack during burial in soil is a common phenomenon which needs to be evaluated too (Arvanitoyannis et al., 1994). The mechanism of biodegradation of the matrix may be covered in three stages: first, the microorganisms grow on the surface of the matrix, then the microorganisms use the matrix material as a source for growth, and finally, destruction of the matrix occurs (Figure 2B; Tharanathan, 2003). Factors such as nutrients, soil temperature, oxygen, pH, and salinity affect the activity and survival of microorganisms (Figure 2C; Rech et al., 2020). The temperature of agricultural soil is reported to be in the range of 18–37°C; for the landfill, the temperature ranged from 18 to 41°C; and finally, the sandy soil showed temperatures between 19 and 34°C. This temperature is close to the optimum temperature for the growth of fungi (22–30°C) and mesophilic bacteria (25–40°C). Soil properties including humidity, phosphorus content, potassium content, and pH were also checked during the biodegradation process. As can be seen in Figure 2C, the PHB films, incorporated with different concentrations of eugenol as an antimicrobial compound, buried in the agricultural soil presented a faster degradation rate than in the other soil types. It was reported that the presence of eugenol did not affect the biodegradation behavior of the films. The high soil moisture content also makes PHB less crystalline and contributes to the increase of the bacterial population (Rech et al., 2020). The bacterial activity is higher in moister environments (Zhang et al., 2016). Overall, the greater the soil fertility, the greater the soil microbial biomass (Rech et al., 2020).

Using natural sources instead of the synthesis materials has been of interest to scientists (Siracusa et al., 2008). Some polymers like celluloses microfibers (CMFs) are hydrophilic in nature, and this property probably helps microorganisms such as fungi and bacteria to penetrate into the matrix using water as an internal environment. It was reported that fungi attack the CMF loaded on the surface of the ethylene/vinyl acetate (EVA) film (Figure 2B; Sonia and Dasan, 2013). This process weakens the polymer matrix and increases hydrophilicity, permeability of the film, and the surface volume ratio. As another example, chitosan exhibited high potential in biodegradable FPS due to its biocompatibility and biodegradability features. The naturally sourced polymers have shown interesting functional features after combination with other materials (Priyadarshi and Rhim, 2020). For instance, the electrospun PVA/chitosan nanocomposite showed good biodegradability along with antimicrobial properties (Pandey et al., 2020). Similar results were reported in a previous study by Yu et al. (2018). In another study, it was reported that the chitosan-based film containing Chinese chive showed a good biodegradability behavior (47.36%) (Riaz et al., 2020). Adding xylan and carvacrol to the chitosan-based films improved both biodegradability and antibacterial activity (Kamdem et al., 2019).

Regarding biodegradable films employed in food packaging, water resistance is a vital property, because in some cases, the packaging will be in contact with humidity and water during the food storage, and due to the high water activity, the packaging’s function will be disrupted. In films made only of polymers (e.g., chitosan, starch, and sodium alginate), higher values for solubility in water at room temperature have been reported, such as solubility rates of 76 and 21% for the chitosan and starch, respectively. It was reported that with the mixture of both polymers without any synthetic polymer (F127 0%), the prepared film showed a solubility of 42% (Fonseca-García et al., 2021), while with the addition of pluronic F127 to the blend of the chitosan–starch, a significant reduction (39%) in the water solubility of the films was reported, which decreased to only 3% when the concentration of pluronic F127 was 5% (Fonseca-García et al., 2021).

In a research, the role of glycerol (as plasticizer) content in modifying the solubility of the material was examined. Thereby, 25% glycerol was added to the starch. The results showed a water solubility lower than that of starch films, and the addition of 30% glycerol resulted in a film with a water solubility of 32% (Loredo et al., 2018). The addition of plasticizers, such as polyols (glycerol), plays an important role in disrupting the interactions between the molecular chains of polymers and weakening them, as well as in increasing the free volume between the chains, for which the reason is the highly hydrophilic nature of the emollient (Sanyang et al., 2015). This enhances the water molecule diffusion into the matrix of films and, finally, increases their solubility.

It has been reported that promoting the mechanical properties of FPS may negatively affect their degradability. However, the mechanical properties of packaging films can be improved by adding a biodegradable synthetic polymer to them (Gómez-Aldapa et al., 2020). Although the prepared polymer is not totally biodegradable, the combination of chitosan with synthetic polymers can otherwise lead to the destruction of nondegradable plastics. Polyvinyl alcohol (PVA), as a nontoxic and water-soluble polymer, is one of the most commonly employed synthetic polymers combined with chitosan. The prepared films not only depicted a highly improved mechanical properties but also promoted barrier performances toward oxygen and water (Giannakas et al., 2020).

**FIGURE 3:**
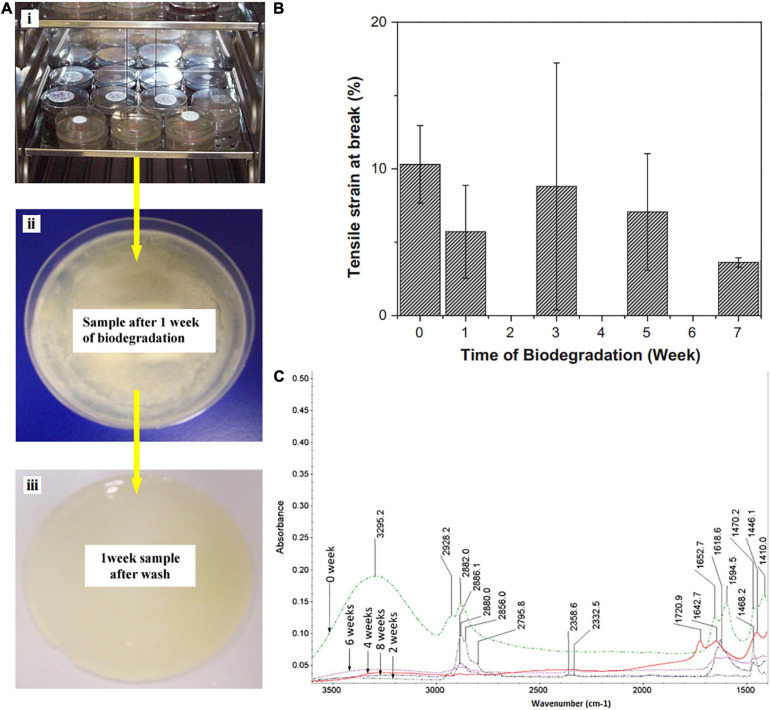
**(A)** Optical view for biodegradation behavior of PVP–CMC films during the biodegradation process in liquid kept inside an incubator at 30°C (i) plates with films in liquid environment inside, (ii) an image of microbial growth on film, and (iii) an image of the film after removing the biodegradation conditions. **(B)** Tensile strain (at break) of dried PVP–CMC films before and after biodegradation. **(C)** FTIR of PVP–CMC films in dry state, before after biodegradation [reproduced with permission from Roy et al. (2012)].

One of the techniques to predict the biodegradation properties of the FPS is a mechanical test. Degradation over time will negatively affect the mechanical properties. In a research, the effect of biodegradation on the mechanical properties of the hydrogel (made of PVP:carboxymethyl cellulose (CMC) (20:80)) was assessed for 8 weeks (Figure 3A; Roy et al., 2012). The tensile strength values of the prepared films were more or less increased with time of biodegradation, and the highest values were revealed after 7 weeks, but during 14 days, the E-modulus gradually reduced because of degradation and enhanced after that. The following reasons were hypothesized for this behavior: modulus E values were initially low due to slow degradation of the polymer film and then increased slowly due to the penetration of microbial growth into the hydrogel film (Figure 3B; Roy et al., 2012). As a packaging substance, it is expected that hydrogel films exhibit higher elongation before break. The hydrogel films should be flexible, not brittle. The dried PVP–CMC films before degradation revealed 10% of strain at break, but as the biodegradation process lasted, the values of strain at break reduces noticeably, and after 7 weeks of degradation phenomenon, the films depicted strain at break of about 4%. This may be due to the reaction of extracellular enzymes secreted by microorganisms in the liquid degradation medium with the PVP–CMC hydrogel and the change or breakage of the pseudo crosslinking bonding structure of hydrogels, which causes a reduction in the values of tensile strain at break (Roy et al., 2012). Figure 3C illustrates the FTIR spectra of PVP–CMC hydrogel for various periods (2, 4, 6, and 8 weeks) of biodegradation. As the degradation began, the peak intensities decreased noticeably. The broad peak at 3,295 cm^–1^ totally disappeared for all the samples during 2–8 weeks (Roy et al., 2012). These significant changes in height and the presence of peaks could be the reason for the decomposition of the hydrogel through some interactions that resulted in a change in their chemical structure leading to biodegradation of PVP–CMC hydrogel films.

In the production of AFP, consideration of environmental conditions can be effective in the process of degradability of packaging coatings. In this regard, the researchers found that the biodegradation time of packaging coatings [e.g., poly(lactic acid) (PLA)] in the environment can be variable and long depending on environmental conditions. Therefore, in order to solve this problem, the addition of organic matter from renewable sources, such as lignin, was suggested, because it can reduce the time of biological degradation. Based on the results, lignin is a suitable choice to accelerate the biological degradation of PLA in garden soil (da Silva et al., 2019).

In conclusion, biodegradable packaging helps the environment remove waste materials left from food packaging. AFP has the high potential of decomposition in comparison with common packaging due to the employed biodegradable materials and embedded specific ingredients.

### Antibacterial Function of Food Packaging

One of the most important food safety concerns in the world is foodborne illness caused by various microorganisms such as viruses, bacteria, and fungi. Some products, such as raw agricultural products, as a major part of food products (e.g., fruits), due to the lack of a way to increase their safety are contaminated by foodborne pathogens (Oliver et al., 2005; Heredia and García, 2018).

Antibacterial property is another vital application in NFP (Table 1). Preventing the growth of microorganisms and consequently food spoilage is one of the main goals of NFP. Increasing consumer demand for organic and healthy foods has led to the use of new technologies for food packaging and storage. Thereby, AFP proved its potential in FPS. Based on AFP, new packaging contains natural antimicrobial agents or is made of antibacterial substrates (Aziz and Karboune, 2018). As can be seen in Table 1 and also based on the published reports regarding antimicrobial properties of different agents, numerous studies have been published about the efficacy of various potent agents against numerous species of both Gram-negative and Gram-positive bacteria, including *Staphylococcus epidermidis*, *Escherichia coli*, *Pseudomonas aeruginosa*, *Staphylococcus aureus*, *Enterococcus faecalis*, and *Vibrio cholera* (Aymonier et al., 2002; Baker et al., 2005; Panáček et al., 2006; Lok et al., 2007; Ahari et al., 2008; Zanjani et al., 2014; Ydollahi et al., 2016; Lotfi et al., 2019; Marrez et al., 2019). AFP is a new candidate for increasing the shelf life and quality of food products (Table 1). According to the “Results” section in Table 1, the efficacy and performance of AFP have been promoted through the nanoencapsulation technique, in which nanoparticles loaded with antimicrobial agents are embedded in the packaging structure, leading to enhancement in food quality during storage (Bahrami et al., 2020).

Incorporation of natural antimicrobial-loaded nanocarriers (metal oxide, essential oil, herbal extracts, etc.) is the most effective way to create AFP in food packaging systems, which has become feasible by employing nanoencapsulated antimicrobials in coating or film structures, instead of interpolation of antibacterial agents (Valdés et al., 2015; Ydollahi et al., 2016; Garcia et al., 2018; Pisoschi et al., 2018; Ju et al., 2019). This approach was further able to make many benefits including release control, protection against environmental stresses, and solubility improvement and natural antimicrobial absorption in AFP, which are the main achievements in overcoming the obstacles of using natural antimicrobials in food packaging (Bahrami et al., 2020).

To promote AFP with the aim of antibacterial power, coating the package with antibacterial agents has been of interest to scientists. The prepared coatings (made of polysaccharide) on paper surface can act as an impressive carrier for interpolation of antibacterial agents. This approach was able further to make the paper materials more practical in AFP (Nechita, 2017). In a research done by He et al. (2021), carboxymethyl cellulose (CMC) film coated with AgNPs was synthesized to promote antibacterial and barrier properties. They reported that CMC-coated paper without AgNPs and the uncoated paper showed no antibacterial activity and no inhibition zone. Contrariwise, CMC paper coated with CNC/AgNPs (cellulose nanocrystals (CNC)) revealed the antibacterial efficacy against *S. aureus* and *E. coli* depending on CNC@AgNPs and the presence of AgNPs. The inhibition power of the coating-based AFP may change against different types of microorganism like species of bacteria. Thereby, it is important to choose the right antibacterial agents according to the food type. For instance, it was found that *E. coli* showed good resistance against the CMC/CNC@AgNPs-coated papers compared with *S. aureus* (Table 2). To synthesize the more efficient AFP, it is important to analyze the structural difference in the cell wall of Gram-negative bacteria and Gram-positive bacteria (Wu et al., 2018). In a similar research, a new nanocomposite has been synthesized using cellulose acetate and AgNPs as antibacterial-based AFP for food safety (Marrez et al., 2019). Based on the minimum inhibitory concentration (MIC) and minimum bactericidal concentration (MBC) results, the new film revealed distinct antibacterial behavior (efficacy) against various types of bacteria, including *S. aureus*, *Bacillus cereus*, *Salmonella typhi, E. coli*, *Klebsiella pneumonia*, with high activity and two strains of *Pseudomonas* spp. with low activity.

**TABLE 2:** Inhibition zone Diameter of coated and uncoated paper exposed to *E. coli and S. aureus* [Reproduced with permission from Wu et al. (2018)].

Samples	Inhibition zone diameter (mm) against:	
	
	*E. coli*	*S. aureus*
Uncoated	0	0
CMC	0	0
CMC/CNC@AgNPs 1%	1.23	2.6
CMC/CNC@AgNPs 3%	2.5	3.8
CMC/CNC@AgNPs 5%	3.3	4.7
CMC/CNC@AgNPs 7%	5.5	6.1

**FIGURE 4:**
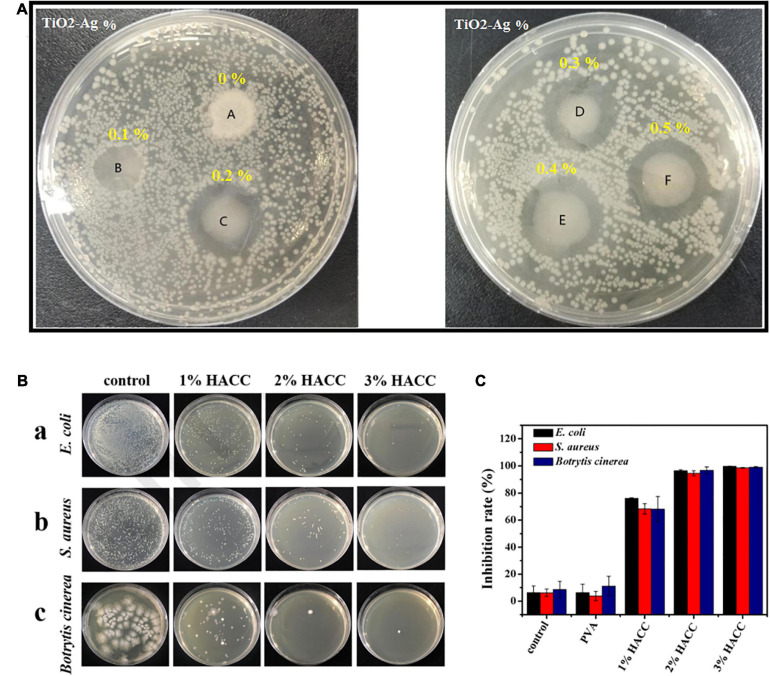
**(A)** Inhibition zone test for fish gelatin–chitosan films containing different percentages of TiO_2_-Ag against *E. coli* [reproduced with permission from Lin et al. (2020)]. **(B)** The antibacterial impact of the chitosan/polyvinyl alcohol (HACC/PVA) composite films: (a) *E. coli*, (b) *S. aureus*, and (c) *Botrytis cinerea*. **(C)** The inhibition rate of different coatings for *B. cinerea*, *S. aureus*, and *E. coli* [reproduced with permission from Min et al. (2020)].

To promote antibacterial-based AFP, apart from AgNPs, other nanoparticles including TiO_2_, CuO, ZnO, nanoemulsions, and nanoclay showed their potential as antibacterial and antimicrobial agents (Table 1). For instance, TiO2-Ag-loaded fish gelatin–chitosan (FG-CH) antibacterial composite was synthesized for food packaging purposes (Lin et al., 2020). FG-CH revealed no antibacterial effect, the reason for which was attributed to the limited antibacterial performance of chitosan at natural pH. After the addition of TiO_2_-AgNp (at the concentration of 0.5%), the film could gain antibacterial ability. These NPs could enhance the UV barrier capability of the film too (Figure 4A). AFP also showed their potential in preventing the coliform bacteria growth during food storage. By employing ZnONP and integration with polymeric films such as low-density polyethylene (LDPE)-based nanocomposite, the coliform bacterial growth was significantly delayed while the neat LDPE could not prevent bacteria (Shankar et al., 2019). In this study, the authors also used grapefruit seed extract (GSE) along with ZnONP to promote antibacterial effects in AFP; thereby, it was reported that the release of flavonoids, which are polyphenolic in structure, might be the reason for the antimicrobial activity of GSE. According to previous studies, flavonoids can diffuse into bacterial membranes and then react with cellular proteins or cytoplasm to destroy bacteria (Corrales et al., 2009; Wang and Rhim, 2016). Generally, the antimicrobial and antibacterial performance of nanoparticles in AFP has been hypothesized to be due to (i) nanoparticle contact with the cell wall of the microorganism directly which damages the bacterial cell, (ii) interference with protein synthesis and the release of Zn^2+^ ions that participate with DNA replication, and (iii) generation of reactive oxygen species (ROS) which degrade bacterial cells (Shankar et al., 2019).

Scientists, to promote more reliable and safer antibacterial-based AFP, employed natural antibacterial agents such as essential oils (EOs) due to their potent antibacterial properties, low price, accessibility, and nontoxicity properties to enhance the shelf life of foods packed with biocompatible materials (Brobbey, 2017; Ju et al., 2019; Sharma et al., 2020a). If such additives were directly incorporated into the food system, a sudden reduction in the bacterial population will be seen, but it may change the smell and taste of food and also limit its long-term efficiency. Various biopolymers capable of holding EO have been employed in antibacterial-based AFP, in the forms of film and coating. It has been proved that embedding EO in the form of nanoparticles will result in better stability and efficacy compared with the free form (El Asbahani et al., 2015).

Regarding their antimicrobial potential, as well as metal nanoparticles, AFP efficacy may be different from different sources, and no single specific mechanism has been proposed for the antimicrobial performance of EO. Antimicrobial potential depends on the concentration of EO, active ingredients, and the type of the microorganisms (Hyldgaard et al., 2012). It was also reported that EO can alter the physiochemical and biochemical aspects of the pathogen (Rao et al., 2019). Studies have shown that the cell wall of Gram-positive bacteria is more sensitive than that of Gram-negative bacteria and is mainly made of peptidoglycan. This makes their walls permeable to hydrophobic compounds such as EOs (Nazzaro et al., 2013).

Another type of antibacterial-based AFP is based on the antibacterial properties of the base material for film or coating production. For instance, chitosan is known as the only natural polysaccharide that shows noticeable antimicrobial activity against a range of microorganisms (Oyervides-Muñoz et al., 2017). Its antimicrobial activity is related to its cationic nature, concentration, deacetylation, exposure time, and organism. The right mechanism of chitosan antimicrobial performance is still unclear. The utilization of natural chitosan was restricted owing to its insolubility in water and antibacterial valency (Oyervides-Muñoz et al., 2017). In another study, as shown in Figure 4B, the ammonium chitosan/polyvinyl alcohol (HACC/PVA) composite coatings revealed significant antibacterial activity for three types of bacteria, while many bacterial colonies were reported in the control groups. Furthermore, the antibacterial efficacy was promoted by enhancement in HACC content (Min et al., 2020) (Figure 4C).

### Indicators of Food Freshness and Spoilage

NFP is looking for new packaging systems to help customers diagnose product quality and spoilage. So far, many studies have been performed to produce packaging systems with the ability to detect corruption. In 20th-century developments in FPS such as packages loaded with oxygen scavengers and antimicrobial agents, new precedents have been established for expanding food shelf-life by protecting them from external environmental conditions.

On the other hand, it would be interesting if consumers realize the quality, nutrition, or spoilage of foods during their shopping without help from specialists. Additionally, if the sellers could easily assess the quality of their products and remove any rotten food from the shelves, the quality of the people’s food basket would increase, and SFP would reveal its potential in fulfilling this approach.

**FIGURE 5:**
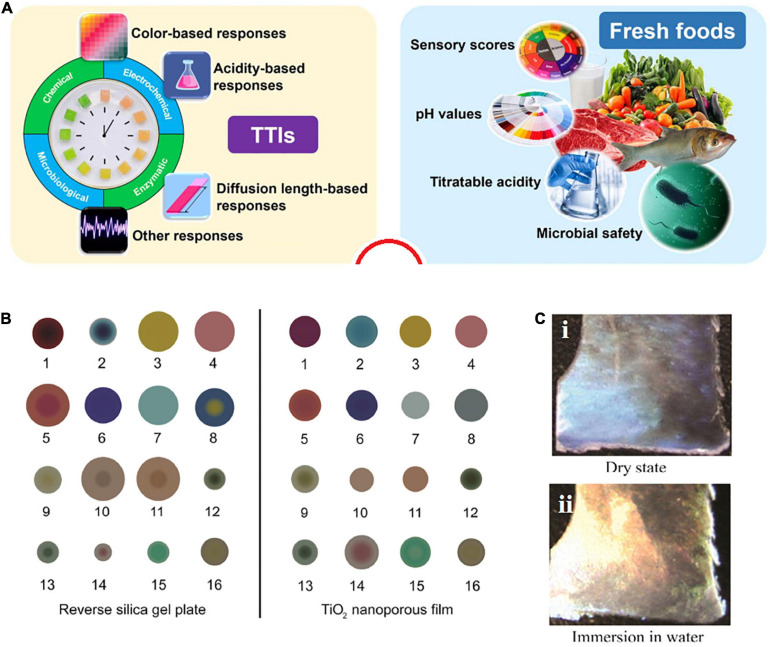
**(A)** Employed parameters for designing TTIs (left) and indexes for informing about the conditions (right) [reproduced with permission from Gao et al. (2020)]. **(B)** A colorimetric sensor based on the TiO_2_ (right) and reverse gel silica plate films (left) [reproduced with permission from Xiao-wei et al. (2016)]. **(C)** Color changes of CNC film: **(i)** dry film, **(ii)** after immersing in water [reproduced with permission from El Asbahani et al. (2015)].

Temperature is a vital factor in distinguishing the shelf life of a food product. One of the strategies for FPS for informing the customer about the status of the product is time temperature indicators (TTIs) (Figure 5A; Gao et al., 2020). These systems are in the form of color dots that exhibit color changes due to temperature changes. These systems are mostly used in products that require specific temperature conditions for storage or consumption (Wang et al., 2015).

In some cases, a product should not be heated above a certain temperature (for example, in a microwave); otherwise, the materials and ingredients of the product will be damaged. In such a case, the color point at the desired temperature indicates a specific color and warns the consumer not to increase the temperature (Aadil et al., 2019). As another example, such a system can be used for some beverage products such as juices. Some products must be stored or consumed at the refrigerator temperature. For the color dot, a special color is considered at the refrigerator temperature, and if the temperature decreases or increases, the color change warns the consumer that the product temperature is not suitable (Aadil et al., 2019). It is very important that color changes be proportional with the real condition of food. For instance, based on Figure 5B, the boundary between color changes is limited, and there is a likelihood of wrong detection. Biological responses depend on biological mechanisms such as spores, microorganisms, or enzymes during specific periods and temperatures (Pavelková, 2013). The Fresh-Check proposed by Lifeline Technologies is another example of a TTI. Its mechanism follows a polymerization reaction and leads to a color change in the indication area. TTIs for spoilage products looks dark in the center, and a clear center means the food is fresh. If the center color matches the color of the outer ring, the product must be consumed soon (Endoza et al., 2004).

Deviations in temperature can cause microorganisms to grow or survive in the food, resulting in spoilage of the product. In general, TTIs or time temperature integrators are simple, inexpensive gadgets attached to the package (Gao et al., 2020). Three types can be distinguished: critical temperature indicators, which show if temperature exceeds proper limits; secondly, partial history indicators, which indicate if a product has been subjected to temperature that causes a change in product quality; thirdly, a full history indicator which records the complete temperature profile along the food supply chain (O’Grady and Kerry, 2008).

The consumer label is another system in SFP. It is a partial-history indicator that provide information about the history of product’s quality based on color changes during storage condition that are different than the recommended storage (e.g., temperature) and will also inform if the product is not consumable anymore (Chen et al., 2019). One of the commercial labels is Timestrips^TM^, which monitors how long a kind of food has been open or has been in use (Food Lable Timestrip, 2020).

There is another type of SFP which is a pH-based system. In a packaged food product, due to the spoilage of the food, the pH level in the product increases or decreases over time, which can be detected by suitable pH sensors. pH sensors change color when exposed to an acidic or alkaline environment, which is a key element of these sensors (Kuswandi and Nurfawaidi, 2017). There are various metabolites which result in pH changes during food storage including glucose (based on glucose oxidases activity), lactic acid (based on lactate oxidase and peroxidase activities), carbon dioxide, oxygen, biogenic amines (based on amine oxidases or transglutamase), and microorganisms. To design pH-based indicators, it is necessary to put the indicator inside the packaging to sense pH changes. The pH-based indicators perform based on color changes as well as TTIs (O’Grady and Kerry, 2008). In this regard, a pH-sensitive color is entrapped within a polymeric network inside the indicator; the acidic liquid diffuses within the network and reacts with the dye; and, consequently, the color changes. The food spoilage (especially fish and meat) correlates with bacterial growth patterns; hence, color changes indicate bacterial growth (Chen et al., 2019).

Considering fruits and vegetables, during ripening, aromas are released by fruits. The novel colorimetric indicators sense the aromas and show various changes in color, whose range depicts the process of ripening (Bordbar et al., 2018). By monitoring the color of the sensor, consumers realize which fruit is at the preferred ripeness. Ripesense^TM^ is known as a commercial indicator with the same protocol (Nanotechnology Products Database, 2020).

Another type of indicators is gas based, which senses the indoor atmosphere of packaging (Weston et al., 2020). During the storage time, some metabolites including CO_2_, H_2_S, O_2_, ethylene, and volatile compounds like ammonia, amines, and ethanol appear in the headspace of the packaging (Mills, 2009; Lang and Hübert, 2011; Koskela et al., 2015; Saliu and Pergola, 2017). These metabolites can be employed as a quality-indicating index for spoilage monitoring by employing an indicator within the packaging system (Mohammadian et al., 2020). Not only do majority of these indicators identify the carbon dioxide and oxygen concentrations (Meng et al., 2014), but water vapor, ethanol, hydrogen sulfide, and other gases are also checked (Fang et al., 2017). A sensor is sensitive and reacts to (gas) changes inside the packaging atmosphere, while an actual indicator detects the quality status. The condition in the atmosphere inside the packaging is based on the food activities, such as chemical reactions or enzymatic actions (e.g., microorganisms generate gases, and the gases transmit through the packaging), and, on the other hand, on the package nature and the storage condition like humidity (Figure 5C).

As the expiration time of different products lasts from days to years, it is important that the SFP keeps its quality at a higher level till the last day of consumption and provide reliable information during storage in the refrigerator or freezer, at normal ambient or even elevated temperatures.

### Scavengers in Food Packaging

**FIGURE 6:**
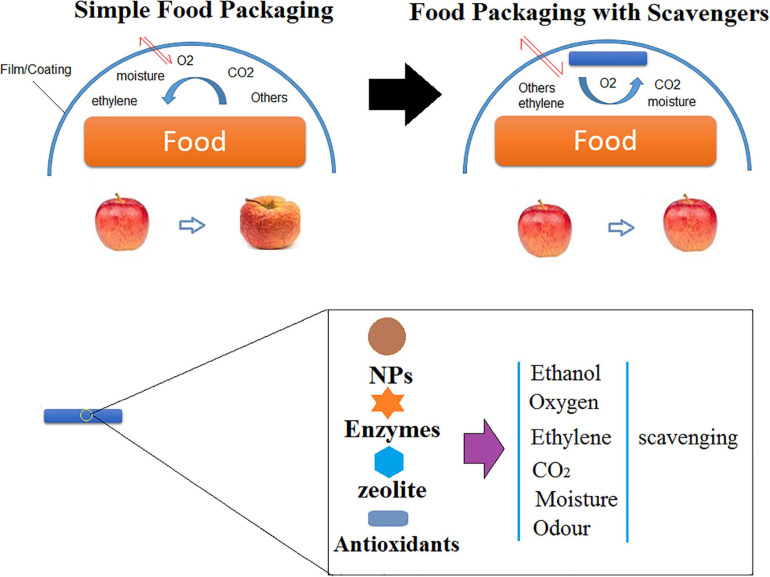
Embedding scavengers in AFP.

NFP includes additives that are capable of absorbing or scavenging carbon, oxygen, dioxide, ethylene, moisture, odor, and flavor taints; releasing carbon dioxide, oxygen, ethanol, sorbates, antioxidants, and other preservatives and antimicrobials; and maintaining and controlling temperature (Figure 6; Biji et al., 2015).

Apart from microbial growth, lipid oxidation is the vital reason for spoilage of various types of foods, including meats, fish, nuts, sauces, milk powders, and oils. It results in a loss of both nutritional and sensorial quality of foods and may even cause the formation of toxic aldehydes. Vacuum packaging is a technique to avoid oxygen accumulation in food packages (Narasimha Rao and Sachindra, 2002). There are some new approaches that are commonly employed to prevent lipid oxidation of packaged foods. In this technique, packaging is under modified atmospheres or direct addition of antioxidants in which the presence of oxygen is limited (Gómez-Estaca et al., 2014). AFPs have been engineered to prevent food spoilage by embedding applicable agents within the packaging (film/coating).

Considering AFP containing oxygen scavengers are able to remove the remaining oxygen in packages, there is a high possibility of affecting the color, taste, and odor of the food and enhancing aerobic bacterial growth before consumption. Various commercialized scavengers that take away the relict oxygen (or other gases) from the packaging are accessible in the form of plastic films, sachets, labels, bottle crowns, and plastic trays (Cichello, 2015). Oxygen scavenging (OS) can employ various mechanisms such as oxidation of iron powder (Miltz and Perry, 2005; Byun et al., 2011). Iron powder sachets are known as the most employed OS agent for commercial purposes. They function based on the criteria of oxidation in the presence of Lewis acids (e.g., FeCl_3_ and AlCl_3_) or moisture (Cruz et al., 2006). In this case, iron powder is embedded within polymer films for AFP purposes. It should be considered that the type of the employed polymer affects the potential OS capacity of iron. The reason turns back to differences in the permeability and barrier properties of different polymeric films (Khalaj et al., 2016).

Other metals including platinum, cobalt, and palladium also revealed OS properties (Cherpinski et al., 2018). It was reported that these metals reinforce the oxygen reaction in the presence of low concentrations of hydrogen, leading to an oxygen level reduction by two orders of magnitude (Yu et al., 2004). Similar to that of iron, their OS efficacy alters with the change in oxygen permeability capacity of the polymers. For instance, different OS efficacy rates for Pd (10% w/w) have been obtained for different polymers. In the case of coating made of palladium + polymer, the oxygen reduced from 1 to 0.28% in 91 min (Pd + silicone rubber), 0.15% in 200 min (Pd + nitrocellulose), 0.31% in 123 min (Pd + polyvinyl butyral), 0.17% in 75 min (Pd + polyurethane), and 0.3% in 60 min (Pd + ethyl cellulose).

The technique of OS comprises stimulation of an inorganic or organic compound embedded in the polymer structure by UV radiation (Dey and Neogi, 2019). 2-Vinylanthraquinone and 2-methylhydroquinone are two organic compounds that when exposed to UV radiation act as oxygen scavengers. An OS system containing triphenylphosphine, 2-methylhydroquinone, and ethyl cellulose decreased oxygen level (from 14.6 to 0.07 vol% every 25.3 h) in the headspace of the container. The triphenylphosphine scavenged the hydrogen peroxide formed during the process (Rooney, 2002).

Unsaturated hydrocarbon-based OS systems are potent candidates for dried foods, but the main problem is the formation of by-products such as ketones, aldehydes, or organic acids which may affect food quality. Organic acids (ascorbic and gallic acids) (Lo Scalzo, 2008), photosensitive dyes (Nie and Xiao, 2020), and enzymes (Hitzman, 1983) are other mechanisms for OS but with lower capacity. Ascorbic acid and α-tocopherol with OS rates of 11.9 and 0.21 cm^3^ of O_2_ day^–1^ g^–1^, respectively, are highly compatible for AFP (Byun et al., 2011; Rodríguez et al., 2020), but both need to be stimulated by UV, light, heat, or transition metals, which entails higher expenses as compared to nano-iron-based scavengers. Besides, they show low efficacy in oxygen scavenging in high moisture content and avoid lipid oxidation because to inhibit lipid oxidation, 98% oxygen scavenging must be obtained (Johnson et al., 2016). The enzyme function of OS is based on reactions with a specific substance. Catalase (Cat) along with glucose oxidase (GOx) are the most commonly employed enzymes for OS in AFP (Gaikwad et al., 2018). According to Eq. 1, to aim for a good OS, it is mandatory that glucose exists in the scavenger formulation or in the food.

(1) $$2\,\text{glucose}+2\,O_{2}+{\text{H}}_{2}\,\text{O}\overset{G\,O\,x}{⟶}\text{D}-\mathrm{glucono}-2\,\mathrm{gluconic}\,\mathrm{acid}+2\,H_{2}\,{\text{O}}_{2}$$

$$2\,H_{2}\,{\text{O}}_{2}\overset{\text{Cat}}{⟶}2\,H_{2}\,\text{O}+{\text{O}}_{2}$$

Embedding clay in the polymeric matrix results in enhancement in OS activity. In this regard, clay prevents glucose pre-oxidation. Besides, the presence of clay in the film matrix increases the porosity of the films, which leads to an increase in surface area and thus an increase in OS capacity (Johansson et al., 2011).

Moisture is another factor which must be controlled in FPS. In this case, moisture scavenging (MS) operatives such as silica gel, natural clays, calcium chloride, calcium oxide, and molecular sieves have been developed to act as OS systems to control residual or excess moisture in packages, thereby preventing or delaying foggy film formation and microbial growth (Ozdemir and Floros, 2004).

To decrease the perishable fruit maturation which happens by the presence of ethylene during a short time, ethylene scavenging (ES) factors are often employed (Gaikwad et al., 2019). The most employed ES agents are based on potassium permanganate; then dispersed minerals like zeolite, pumice, and active carbon; and finally, metal catalysts like palladium with charcoal (Álvarez-Hernández et al., 2019).

Carbon dioxide is another factor which needs to be controlled for increasing food shelf life. Carbon dioxide scavenging (CS) agents, including zeolites and calcium hydroxide, are mostly employed to remove CO_2_ gas generated from microbial growth, degradation mechanisms, and respiration. CO_2_ releasers such as food acids and sodium bicarbonate decrease the microbial growth and the respiration rate of vegetables (Lee, 2016). Different synthetic antioxidants including organophosphates and polyphenols and natural antioxidants like spices and herbs are embedded in the packaging to delay or prevent the negative effects of oxidation and thermal degradation (Lee, 2016). To sum up, scavenging-based AFP, such as NFP, is a potent candidate to reduce lipid oxidation and food spoilage in packaged foods (Gómez-Estaca et al., 2014).

## Biomaterials for NFP

Since 50 years ago, plastic has been widely used in the production of packaging materials due to its ease of production, storage, and transportation. With the development and progress of the food industries, there is a great demand for packaging materials based on petroleum products such as PVC, PET, polystyrene (PS), polypropylene (PP), and polyamide (PA) for use in this industry (Ebnesajjad, 2012). Despite their advantages, these plastic products have caused serious environmental problems due to their nondegradability in the environment (Rhein and Schmid, 2020).

The advent of biopolymers is a good solution to improve the environmental problems caused by plastics, because they are biodegradable despite imitating the properties of plastics and only remain in the environment for a short time. Biopolymers are divided into three categories based on the source of production (Table 3; Gupta and Dudeja, 2017) polymers extracted from biomass (Grönman et al., 2013), those synthesized from biomass-derived monomers, and (Muncke, 2014) those produced from microorganisms (Mangaraj et al., 2014; Naveena and Sharma, 2020). Considering applications in the food industry, these bio-based materials are particularly applicable in three main areas in FPS: food coating, food packaging, and edible films. NFPs basically employ biomaterials from the category above for professional purposes.

**TABLE 3:** Three category of bio-based polymers.

Bio-based Polymers				

Polymers extracted from biomass			Polymers synthesized from bio-derived monomers	Polymers produced from microorganisms
		
Polysaccharides	Proteins	Lipid	Polylactate	Bacterial compounds
Starch Chitosan Chitin Potato Corn Wheat Rice Other derivative	Animal Casein Whey Collagen Gelatin	Waxes Oils Fats	Polylactic acid Polycaprolactan	Cellulose Xanthan Culanpullulan
Cellulose Cotton Wood Other derivative	Plant Zein Soy Gluten			
Gums Guar Locust bean Alginate Carrageenan Pectin				

In order to use these materials, it is necessary to produce them in a specific form (polymeric films). There are various film-forming methods for biopolymers, including the melt mixing method, solution casting method, thermal pressure method, electrospinning method, and extrusion blown film method. The quality of polymers can be described as various properties such as physical, thermal, mechanical, and barrier properties. With increased awareness on sustainability, packaging industries around the globe are looking for biopolymers as a replacement for synthetic polymer. Biopolymers may be defined as the polymers that are biodegradable by the enzymatic action of microbes. In the last two decades, a lot of research has been done on biopolymers for food packaging applications (Fabra et al., 2014).

The employment of edible packaging (EP), as a thin layer covering the food surface, drew much attention from scientists and industries (food and beverage). EP has the ability to remove the issues associated with plastic packaging systems. EP showed several targets including moisture loss restriction, gas permeability management, quality of microbial activity (e.g., chitosan, which acts against microbes), gradual release of flavors, and maintenance of the structural integrity of the product in the food (Jeevahan and Chandrasekaran, 2019). However, the sources of biopolymers used for NFP are also important.

**Group 1:** Polymers directly derived, removed, or extracted from biomass showed reliable results in FPS. Cellulose, starch, and proteins such as casein and gluten, as certain polysaccharides, are a few of the commonly used biomaterials. However, all of them are inherently hydrophilic and crystalline, causing problems during processing. In case of moist foods, their performances revealed poor results, whereas due to their excellent gas barrier properties, they are a good candidate for FPS (Azeredo et al., 2014). This type of biomaterials is useful for expanding the shelf life.

Other extracted materials from biomass resources, like polysaccharides (e.g., chitosan), proteins (e.g., zein), and lipids (e.g., waxes), also showed excellent potential as aroma and gas barriers in FPS. Chitosan has depicted significant potential as an antimicrobial biomaterial to keep foods from different microorganisms (Wang H. et al., 2017; Mohamed et al., 2020). In a research, pork sausages were covered by chitosan film, resulting in oxidative and color stability improvement (Siripatrawan and Noipha, 2012). Previous studies focusing on AFP have assessed the antimicrobial efficacy of chitosan when combined with other polymers, such as starch (Haghighi et al., 2020). Green plants such as rice, wheat, corn, and potatoes are the most common and new ingredients employed to produce starch biopolymers. It was reported that when chitosan is combined with tapioca starch, greater antimicrobial effectiveness was observed (Vásconez et al., 2009). Starch due to its low cost and availability is known as the most preferred biopolymer. Cellulose, with potent mechanical features and being made of glucose units, is a polar polymer. The configuration of the polymer chain in a different way from that seen in starch provides an opportunity to strengthen this polymer due to the formation of strong hydrogen bonds (Chen et al., 2014).

The main disadvantages of these kinds of materials are the inherently high adversity and problem of processing them in common and conventional equipment (Zhao et al., 2019). For instance, although starch is known as a biodegradable biopolymer that can be synthesized in large quantities with low cost, can be easily handled, and can form films for FPS with low oxygen permeability, the main challenge of native starch is that it is fragile and hydrophilic. This weakness fails them in NFP. These issues restrict its different applications like its employment for plastic bags and food packaging manufacturing (Weber et al., 2002). To overcome this drawback and improve its flexibility and ease of processing, various plasticizers like glycerol, glycol, and sorbitol are utilized to make the starch into a thermoplastic starch (TPS) using heat and extrusion processes (Abdorreza et al., 2011; Isotton et al., 2015; Mikkonen et al., 2015). In the case of microencapsulation and nanoencapsulation in NFP, these biomaterials showed their potential too. For instance, encapsulation of probiotics, antioxidants, and bioactive ingredients help NFP to act as an AFP. Each of these ingredients, due to their chemical structure and surface charge, needs specific biomaterials to be encapsulated with. The most widely employed material for probiotic encapsulation is alginate, which can be employed alone or combined with other biomaterials like chitosan (Chandramouli et al., 2004).

Another disadvantage of polysaccharides and proteins is their very potent water sensitivity generated by their hydrophilic nature (Chivrac et al., 2009). This leads to a powerful plasticization resulting in oxygen barrier properties being spoiled as the water sorption and relative humidity in the matrix of materials increase (Laufer et al., 2013). The low water resistance of proteins and polysaccharides restricts their usage in FPS. It would be extremely advantageous to decrease the water sensitivity of polysaccharides and proteins and to improve the gas barrier properties and total functionalities of thermoplastic biopolyesters to promote their properties in APS (Laufer et al., 2013). Proteins, based on their origin, comprise two categories, namely, plant proteins (e.g., gluten, soy, potato, zein, and pea) and animal proteins (e.g., casein, collagen, whey, and keratin) (Ahmadzadeh and Khaneghah, 2019). Proteins, due to their functionalities, are suitable to be modified to make a polymer. Protein-based polymers (except keratin) are sensitive to moisture. This sensitivity can be overcome by lamination or blending with chitin (as the second abundant polysaccharide). Chitin is abundant in marine invertebrates like crabs, shellfish, shrimps, insects, and some yeasts and fungi. Chitosan and chitin are eco-friendly, nontoxic, water-insoluble, and biodegradable polymers, with no antigenic features; besides, they show good biocompatibility (Priyadarshi and Rhim, 2020). Lipid compounds such as wax and glycerides are mostly employed to provide hydrophobicity in FPS (coating/films). They impressively prevent water vapor diffusion within edible films. This ability will help FPS to prevent the growth of microorganisms and food spoilage as a feature of AFP. The functional characteristics of FPS containing lipids are efficiently influenced by lipid properties including the physical state, saturation degree, structure, chain length, the crystal morphology, and distribution. Isolated soy protein-based films are moisture sensitive, which can be modified by the incorporation of stearic acid (SA) in a proportion of 25% (Lodha and Netravali, 2005; Han Y. et al., 2018). It has been recently revealed that incorporation of soy protein with K-carrageenan, glycerol, or gellan gum is effective in creating edible and biodegradable soy protein-based FPS.

**Group 2:** Synthesized polymeric materials are produced by classical polymerization techniques like aliphatic polyesters, aliphatic aromatic copolymers, aliphatic copolymer, polylactide using renewable bio-based monomers including PLA, and oil-based monomers such as poly-caprolactones (PCL). PLA is a good and well-known example of polymers and can be created into injection mold objects, blown film, and coating. PLA is popular in FPS due to its supreme transparency and relatively noticeable water resistance. In a study, PLA in combination with PCL, loaded with carvacrol and thymol oil, could show antioxidant activity as an AFP (Lukic et al., 2020). In another research, PLA films were functionalized to transfer cinnamaldehyde as an AFP (Villegas et al., 2019). The main challenge for these biomaterials is to promote their thermal and barrier properties; thereby, they act like PET. PLA can be used as laminates (barrier film) or homo-materials for FPS. However, it is not pliable, and it is also sensitive to rupture. PCL as another one of the most employed polymers in FPS is a hydrophobic aliphatic polyester which can be produced by chemical reactions from either renewable resources like polysaccharides or petroleum. PCL with a low viscosity is a biodegradable, thermoplastic, and biocompatible polymer, which has a low glass transition temperature. Besides, it can easily be utilized with common melt processing equipment (Cabedo et al., 2006; Woodruff and Hutmacher, 2010; Rešček et al., 2015). PCL in NFP depicted an antimicrobial character, inhibition or quenching potential, spoilage prevention, pathogenic microorganism removal, and at same time being environmentally friendly (Pina et al., 2020). There are other synthesized biopolymers that showed similar potential in NFP. Poly(vinyl alcohol) (PVA) has been another polymer suitable for food packaging. The films or coating prepared by PVA showed that it can be a good candidate in transferring antibacterial agents while controlling moisture, oxidation, and sensory properties (Bhat et al., 2021). poly(lactide)/poly(butylene adipate-co-terephthalate) (Sharma et al., 2020b), poly(methyl methacrylate) (Lin et al., 2019), and poly(*l*-glutamic) acid–poly(*l*-lysine) (Karimi et al., 2020) are some more examples that are employed in NFP due to their ability to provide antibacterial activities, to control nanoparticle migration, and to have good mechanical behavior and biodegradability.

Isocyanates and polyol are utilized to synthesize films/coating suitable for FPS. Polyol can be generated from edible oils, and isocyanate can exclusively be generated from petroleum-based feedstock (Dong et al., 2020). Diversified synthesis and working on the parameters can give various biomaterials different characteristics.

**Group 3:** Polymers which are synthesized by genetically modified bacteria or microorganisms showed their potential in FPS. Different types of microorganisms such as *Bacillus*, *Alcaligenes*, *Rhizobium*, *Halobacterium*, and *Azotobacter* produce large amounts of renewable and biodegradable materials. These substances are collected by bacteria as an energy source and a store of carbon. Bacterial cellulose (BC) and polyhydroxyalkanoates (PHAs) are two of the main polymers which are synthesized by microorganisms and are highly used in FPS (Ivonkovic et al., 2017). PHAs as hydrophobic polyesters may have various properties and characteristics related to monomer building blocks, which lead to a variety of PHA types. The most important PHAs are PHB and the copolymers poly(3-hydroxybutyrate-*co*-3-hydroxyvalerate) (PHBV) and poly(3-hydroxybutyrate-*co*-3-hydroxyhexanoate) (PHBHHx) (Gouvêa et al., 2018). PHBV, as well as other biomaterials, showed its potential in NFP by carrying nanoclay functionalized by oregano EO. It showed better oxygen barrier properties with higher antimicrobial activity (da Costa et al., 2020). PHA may be a rigid, brittle, or rubber-like polymer depending on the carbon source and the bacterial type (Sharif et al., 2020). Copolymerization is one of the techniques to improve physical–mechanical properties of PHAs as well as other techniques such as blending. This behavior makes it a good candidate for various purposes in NFP proportional to the food type. BC has been employed for a number of distinct applications, specifically in the biomedical area. Although there are several noticeable applications of BC in FPS, only a few studies reported on this area, the reason for which, as a limiting factor, is the expensive process of BC production (Azeredo et al., 2019). BC in combination with chitosan could act as a biodegradable film for active packaging materials (Xu et al., 2021). Based on the results, the prepared film depicted the best antioxidant activity. In a research, it was reported that more than 50% of pure BC film goes through degradation in 3 days and 100% after 7 days in the soil (Zahan et al., 2020). Interestingly, *Bacillus* sp. and *Rhizopus* sp. were identified as the bacteria responsible for BC degradation.

In case of coating foods, under direct coating, the main goal is immobilization of the polymer directly on the food surface to provide adequate protection (due to containing active substances) from environmental hazards like microbes. Also, the coating can alter the gas permeability coefficients of the fruits and vegetables, thereby altering their respiration rate and shelf life. These coatings are environmentally friendly, biodegradable, and, in most cases, edible. Overall, three types of coating methods have been explored: spread coating, spray coating, and dip coating.

## Nanotechnology as NFP Material

Subsequent generation of materials employed in NFP is presumably bio-nanocomposites. Expansion of bio-nanocomposites is a direction to develop original and inventive biomaterials in the area of NFP. Nowadays, numerous nanoscale substances (metal nanoparticles, polymeric nanoparticles, nanoemulsion, nanoclay, and so on) are candidates in food packaging systems including AFP and SFP. Incorporation of nanotechnology with FPS enhances the viability and stability of susceptible active compounds and promotes the essential packaging functions—preservation and protection, containment, communications and marketing, and the efficacy of FPS.

On one hand, nutritional features of packaged foods can be promoted by utilizing nanoscale additives and nutrients and delivery systems for bioactive compounds (Silvestre et al., 2011). On the other hand, to enhance food shelf life and prevent spoilage, different functional agents like antimicrobials, antioxidants, colorants, anti-browning agents, and enzymes can be employed in FPS in the nano dimension by embedding them into the polymeric matrix or coating them on the surface (Kanmani and Rhim, 2014; Alfei et al., 2020).

One of the main aims in NFP is improving the packaging properties including film/coating flexibility, gas barrier properties (e.g., carbon dioxide, moisture, oxygen, and emission of ethanol and flavors) (Mei and Wang, 2020), and temperature/moisture stability (Kraśniewska et al., 2020). Employing diverse NPs in FPS is an option to boost these improvements. Thereby, integration of FPS and NPs has developed various polymeric nanocomposites and polymeric nanomaterials (Alfei et al., 2020). These nanomaterials may be employed alone or along with other NPs for FPS development. In this case, to promote and maintain texture, flavor, and color; reduce corruption; prevent adulteration; and enhance stability during storage, diverse nanofillers can be used including nanoclay, silicate, carbon nanotubes, graphene, metal NPs and their oxides, nanocellulose, and EO-based nanoemulsions (Carbone et al., 2016; Han C. et al., 2018; Biswas et al., 2019; Espitia et al., 2019; Emamhadi et al., 2020). NFP with the help of NPs has also more active properties such as biocatalysts and antibacterial actions (Kim et al., 2020). In order to make optimal use of NPs, they must be obtained completely homogeneously in the polymer matrix because the degree of homogeneous dispersion of NPs in the polymer matrix alters the relaxation of the polymer chains and molecular mobility, subsequently affecting mechanical properties and thermal resistance. Also, NPs also provided an interesting potential for NFP by which they are planned to act as gas scavengers, sensors, and condition indicator (food and environment surrounding) or can also serve as a protector against fraudulent imitation (Ding et al., 2020). As previously mentioned, there are numerous NPs which are employed in NFP [this topic has been nicely reviewed by Alfei et al. (2020)].

### Nanoclay NP in NFP

One of the most employed NPs is nanoclays that are aluminum silicate made of fine-grained minerals with a natural structure in sheet-like geometry. Nanoclay for NFP is utilized in the polymer formation as nanocomposites due to its high stability and benignity, convenient process ability, low cost, and good availability. Montmorillonite (MMT) is a clay nanomaterial emanating from volcanic or rocks ash, which is extensively employed in nanocomposites (Bai et al., 2020). When nanoclay is embedded in a polymer matrix, they oppose the penetration of gases and other materials. Nanoclay NPs showed their potential in improving barrier properties and minimizing gas and small-molecule transmission between food and the outside packaging environment, which shows improvement in diffusion and solubility coefficients (Horst et al., 2020).

### AgNPs in NFP

AgNPs as an inorganic metal oxide are industrially produced by mechanochemical or physical vapor methods (Casey, 2006). The use of AgNPs in NFP has recently attracted much attention. AgNPs possess the most impressive antibacterial properties against a wide range of microorganisms such as yeasts, bacteria, viruses, and fungi (Moadab et al., 2011; Martínez-Abad et al., 2012). Besides shelf life enhancement, AgNPs cause no change in food physical characteristics (Ahari, 2017). Numerous studies reported about the potential of AgNPs in NFP for orange juice and beverage (Emamifar et al., 2010), bread (Sattari et al., 2010), fish and meat (Umaraw et al., 2020), and fruits and vegetables (Ijaz et al., 2020). AgNPs showed improved potential when combined with other nanoparticles such as ZnO and CuO.

### ZnONP in NFP

The application of ZnO nanoparticles increased in safe food packaging due to their antimicrobial activities, UV blocking, and being cheaper than AgNPs (Emamifar et al., 2010). By integrating ZnONPs with polymeric FPS, characteristics such as mechanical strength, blockade properties, and durability can be noticeably improved (Espitia et al., 2012). It was reported that the uniformly dispersed ZnONPs within the PLA matrix effectively adjusted the diffusion of penetrant molecules (e.g., CO_2_ and O_2_). The reason was increasing the tortuosity of the diffusive route (Vasile et al., 2017). Similar results were reported by Sossio Cimmino. They also reported the high potential of ZnONPs in preventing *E. coli* growth (Marra et al., 2016). As previously mentioned, the proposed mechanisms for microbial growth prevention by NPs comprise cell respiration, cell wall disruption, and interaction with thiol groups of DNA and sulfhydryl or phosphorous, proteins and enzymes, and ROS (Kanmani and Rhim, 2014). For example, generated ROS due to NPs causes disruption of the cell membrane, damaging the DNA and mitochondria and interrupting transmembrane electron transport in the cell (Garcia et al., 2018).

### TiO_2_NP in NFP

TiO_2_, CuO, and AlOx, as well as ZnO, AgNPs, and silica, are also employed in FPS. These NPs (in the form of being blended with or coated on the diverse biological and synthetic polymers) are generally employed as photocatalysts with antimicrobial and EE properties and can also improve mechanical properties (e.g., tensile strength) and gas barrier and UV barrier properties of the FPS (Xing et al., 2012; Bumbudsanpharoke et al., 2015). There are several reports that bond formation between TiO_2_NPs and the polymer prevents the interaction of water molecules with the polymeric chains by blocking the number of sites in the polymer chains (Siripatrawan and Kaewklin, 2018; Anvar et al., 2019). It was also found in studies that addition of TiO_2_NP to the polymeric films increased the tensile strength and elongation at break (Xing et al., 2012).

### SiO_2_NP in NFP

Modification of nano-SiO_2_ with ethylene/vinyl acetate and mixed with polypropylene (PP) showed tensile strength enhancement and gas permeability improvement. Compared to the pure nanocomposite, addition of SiO_2_ resulted in less ink solvent adsorption, which can be important in the case of laminated food packaging as the PP layer is used for printing (Li et al., 2016). SiO_2_ also showed its potential in adjusting the barrier properties of coated nanocomposites. For example, this process increased the gas barrier properties up to 70% compared with the pure PLA films while keeping its physical properties (e.g., remaining transparent) (Bang and Kim, 2012). In addition, silicon is one of the most widely used nanoparticles in the fight against Gram-positive and Gram-negative bacteria and fungi. Its antimicrobial effect in a variety of food packaging coatings has been proven in recent studies (Al-Tayyar et al., 2020; Hajizadeh et al., 2020).

### AlOxNP in NFP

Aluminum oxide (AlOx), as well as the previous NPs, has revealed high potential in FPS in the viewpoint of barrier properties. PLA-Al_2_O_3_-coated board paper, compared with the double-coated PLA film with a layer of Al_2_O_3_ and a layer of alginate–chitosan, substantially increased the water, oxygen, and aroma barrier properties (Hirvikorpi et al., 2011). In another study, it was reported that coating of PET with aluminum oxide could also promote the barrier properties, which could become a potent substitute to the common metallized retortable packaging (Struller et al., 2014).

### SnO_2_NP in NFP

Nanocrystalline stannic oxide (SnO_2_) showed its potential in indicator-based NFP. SnO_2_ has the potential to indicate oxygen because a dye photo-reduction can change the film’s color depending on oxygen exposure. This system would be beneficial to approving the effectiveness of vacuum or nitrogen packaging. There are other NPs with similar performances but different efficacies [nicely reviewed by Jildeh and Matouq (2020)]. There are more reports about the antibacterial feature of SnO_2_NP. Amininezhad and his colleagues reported that SnO_2_NPs depict notable antibacterial activity against both Gram-positive and Gram-negative bacteria (Amininezhad et al., 2015). It was also reported that they had higher activity against *E. coli* than against *S. aureus*. In another study, scientists showed the antifungal properties of SnO_2_ activity against *Candida albicans* (Fakhri et al., 2015).

### Nanoemulsion in NFP

Other types of nanosystems such as nanoemulsion showed high functionality in NFP. Nanoemulsions possess nanosized droplets. This colloidal systems, due to the small size of the droplets, are kinetically stable, and their formulation comprises two immiscible liquids (water and oil) and an emulsifier [nicely reviewed by Salome Amarachi et al. (2014)]. The structure and composition of this system can be engineered for the loading, encapsulation, and fruitful delivery of antimicrobial, oxygen scavenging, antibacterial, antioxidant, and flavoring and coloring agents (Salem and Ezzat, 2018). Based on the literature survey, the thermal, mechanical, barrier, and sensory properties are the most studied topics in nanoemulsion-incorporated NFP. In nanoemulsions, plant EO and oil compounds are employed as dispersed phases which are revealed to be useful antibacterial agents with an anti-plasticizing role in NFP (Du et al., 2008; Ahari and Massoud, 2020). The incorporation of EO into NFP caused higher water vapor permeability values and gas permeability in packages that possess low water barrier properties (Dammak et al., 2017). Furthermore, nanoemulsion-based NFP depicted remarkable antimicrobial activity when one of the components of the nanoemulsion is a biologically active agent. For instance, scientists reported that methylcellulose-based packaging films incorporated with oregano or clove bud EO enhanced the shelf life of sliced bread (Otoni et al., 2014). Salvia-Trujillo et al. (2015) expanded sodium alginate-based edible coatings loaded with lemongrass EO-based nanoemulsions to preserve freshly cut Fuji apples. In another study, thyme EO nanoemulsions showed their antibacterial effect on fish as a component of AFP systems, resulting in bacterial cell membrane rupture (Ozogul et al., 2020). Alexandre et al. (2016) integrated ginger EO with gelatin for food packaging application. The final film showed good antioxidant activity. Employing the nanoemulsion of saffron showed good antibacterial features in food packaging systems. The results of Ahari and his colleagues, as a patent, depicted high potential of saffron nanoemulsion in NFP (Hamed et al., 2021). *Thymus daenensis* EO embedded in hydroxypropyl methyl cellulose edible films could improve antibacterial activity and physical and mechanical properties (Moghimi et al., 2017). In addition, employing nanoemulsion enhanced the bioactivity and caused reduction in the impact on organoleptic characteristics.

Pickering emulsions are another new method in the field of nanoemulsions that has attracted a lot of attention in the field of food science (Chen et al., 2020). Unlike conventional nanoemulsions and emulsions, stabilization of Pickering emulsions is made by solid particles, which can be irreversibly absorbed at the oil–water interface and form a dense film (made of solid particles) to prevent droplet accumulation. Compared to conventional emulsions, Pickering emulsions have the advantages of less usage of emulsifiers, biocompatibility, higher safety, higher stability, reduction of droplet aggregation, and uniform droplet size distribution. Different types of solid particles include chitosan (Lim et al., 2020), starch (Zhu, 2019), cellulose (Liu et al., 2019), whey protein (Zhang Y. et al., 2020), zein (Zhu et al., 2019), soy protein (Ju et al., 2020), fat crystals (Wang Q. et al., 2017), and hydroxyapatite (Rodriguez et al., 2019) [nicely reviewed by Chen et al. (2020)]. Food-grade Pickering emulsions have significant applications in food packaging. For example, stabilized starch nanocrystal Pickering emulsions have been used to produce starch nanocrystalline nanocomposites, which are employed to produce active packaging coatings with better optical and mechanical quality (Zhu, 2019). A similar study was done by Almasi et al. using WPI–inulin complexes for stabilizing. The final film showed good mechanical behavior (Almasi et al., 2020). In another research, Pickering emulsions were prepared using encapsulated hesperidin and then stabilized by chitosan nanoparticles. The obtained Pickering emulsions were integrated with gelatin to produce films/coatings with good flexibility while having strong antioxidant activity (Dammak et al., 2019).

Based on the results, an impressive antimicrobial behavior of the nanoemulsion-based coating was observed against *E. coli* compared to the control group. To sum up, scientists utilized different types of nanomaterials to promote NFP to aim in SFP and AFP (Nasiri et al., 2019).

## Hazards of Nanotechnology in NFP

Although the nano-based SFP and AFP under the category of NFP benefit from novel systems including nanoemulsions and nanoparticles, various reports have revealed many uncertainties still remaining about these systems, such as their interest for bioaccumulation, migration to food matrix, toxicity of the biomaterials, and human health risks. On one hand, scientists try to synthesize NFP, and on the other hand, many of them are worried about the consequences. Several commercialized forms of nano-based NFP in the form of composite or coating containing inorganic materials are employed in FPS. Some of them contain AgNPs, CuNPs, TiO_2_NPs, and other metal NPs as the main agent for preventing microorganism activity (Brobbey, 2017; Aziz and Karboune, 2018; Bahrami et al., 2020). Numerous studies have reported about the possible migration of nanomaterial from FPS to foodstuff (Song et al., 2011; Ahari and Lahijani, 2021). There is still a debate among researchers about the extent to which migration is negligible and safe (Bumbudsanpharoke and Ko, 2015). A group of scientists have indicated that nanoparticles such as AgNPs have the capacity to damage human cells. This damage may happen by rectifying the function of the mitochondria, producing (ROS), and enhancing membrane permeability (Song et al., 2011). Scientists have reported that time and temperature are effective in nanoparticle migration. For example, it has been assessed that as time and temperature increased, AgNP migration slightly enhanced in 3% (w/v) acetic acid before reaching a steady state (Song et al., 2011). In a similar research, it has been reported that generally, the level of NP migration is significantly enhanced by temperature and time in all food-simulating solutions (Huang et al., 2011).

The conceivable mechanism considered for the NP migration phenomenon comprises two steps. First, the initial release is considered to be from the encapsulated NPs located on the specimen surface layers. Then, the further release of NPs is accomplished by the geminate-sorption process, embedding, and diffusion. Along with metal NPs, the migration of other types of nanomaterials like nanoclay, nanoemulsion, and microcrystalline cellulose has been monitored. Various polymeric nanocomposites (e.g., PE, PLA, and LLDPE) are integrated with one type of nanomaterials (e.g., nanoclay, silver, copper, or iron) with purposes of SFP and AFP. When they went into contact with real foods or food simulants, NP migration could not be neglected, although different results have been reported. It means that choosing materials with the least migration capacity is a criterion in NFP (Huang et al., 2011).

Apart from nanoparticles, monomer migration has been reported and confirmed as a hazard in NFP. In this case, Pilevar et al. (2019) reported about the migration of styrene monomer (SM) from packaging made of polystyrene into foods. Based on the results, the characteristics of polystyrene packaging material in the viewpoint of their residual SM level and the storage conditions of foods can greatly affect SM migration. Also, food characteristics such as pH, moisture, and fat content can dramatically affect SM migration. Scientists showed their report to prevent or lower SM migration; for instance, in a research, organoclay and zinc oxide nanoparticles (ZnONPs) were employed for the control and optimization of the SM migration into food simulants. It has been concluded that these NPs reduced SM migration but not completely. As another concern about the entrance of NFP to human life, migration of chlorinated paraffins () is one of the main hazards. Wang et al. (2019) reported the migration of CP from plastic food packaging into food simulants. In different types of NFP, multilayer material food packaging (20 multilayer) employed in FPS is generally manufactured with a polyurethane adhesive layer in its structure, which may include cyclic ester oligomers as hazard migrants in FPS (Ubeda et al., 2020). The results showed the migration of cyclic ester oligomers into stimulants.

Consequently, there are studies which report about the migration of active agents (like nanoparticles) in NFP (SFP and AFP), which means there is still a need for sufficient toxicological data about the safety assessments of NFP. Thereby, more research is mandatory to promote NFP and ensure that NFP does not cause hazard to human life.

## Conclusion and the Future Prospective

The aim of this review was to discuss the NFP systems including AFP and SFP talking about their main functions, features, and components. NFP could show high potential in functions like antibacterial function, degradation, indication, and scavenging. These functions can be designed or controlled depending on the food type. Surprisingly, to the knowledge of the authors, AFP systems showed significant progress in shelf life enhancement by controlling the microorganism growth, relative humidity, oxygen level, barrier properties, and humidity. Employing different kinds of biomaterials, nanoparticles, nanoemulsions, and different carriers improved these functions. Making SFP has been another brilliant progress in NPS. It would be interesting if the consumer is aware of the product quality during their shopping without any aid from an expert. The embedded indicators and sensors can react against different environmental changes in the food matrix or inside the packaging such as pH, humidity, and temperature. Embedding sensors and indicators in FPS can alert the consumer about the food quality by a range of colors. Regarding environment issues, biodegradable packaging materials can notably decrease the packaging residual in the environment. Reaching a smart packaging that is environmentally friendly and with high mechanical quality, no toxicity, and low cost still needs more studies. It seems that the industry of packaging is going to experience a revolution in FPS.

In spite of the remarkable advantages of NFP and the employment of various technologies, scientists still warn about the side effects and hazards of the employed changes in FPS. There is still a gap between the ideal food packaging system and the current situation. Addition of nanoparticles as a remedy to fight against microorganisms, prevent their growth, act as an indicator of quality and spoilage, and act as a gas scavenger improved the quality of FPS, while there are evidences that it increases the likelihood of nanoparticle migration from the packaging to the food matrix. Importantly, the embedded sensors or indicators in SFP should guarantee their accuracy and validity each time from packaging until consumption. They should not be sensitive to other parameters like environmental conditions (temperature, humidity, pressure, and so on). In general, the results of some studies still recommend more research to guarantee the future of NFP.

In conclusion, there has been a promising progress in FPS, and people will experience a healthier life than before, but it is important to carry out adequate studies to reach a more reliable FPS. This progress should not affect the final cost of products.

## Author Contributions

Both authors announce the equal contribution in literature review and the manuscript preparation.

## Conflict of Interest

The authors declare that the research was conducted in the absence of any commercial or financial relationships that could be construed as a potential conflict of interest.
